# Distribution characteristics of the *sabA*, *hofC*, *homA*, *homB* and *frpB-4* genes of *Helicobacter pylori* in different regions of China

**DOI:** 10.1371/journal.pone.0268373

**Published:** 2022-05-19

**Authors:** Mengyang Fang, Zhijing Xue, Lihua He, Yuanhai You, Yanan Gong, Dongjie Fan, Lu Sun, Kangle Zhai, Yaming Yang, Jianzhong Zhang

**Affiliations:** 1 Department of Epidemiology, School of Public Health, Nanjing Medical University, Nanjing, China; 2 State Key Laboratory of Infectious Disease Prevention and Control, Collaborative Innovation Center for Diagnosis and Treatment of Infectious Diseases, Chinese Center for Disease Control and Prevention, National Institute for Communicable Disease Control and Prevention, Beijing, China; Northwestern University Feinberg School of Medicine, UNITED STATES

## Abstract

**Background:**

*Helicobacter pylori* (*H*. *pylori*) encodes numerous outer membrane proteins (OMPs), with considerable geographic heterogeneity and related to different clinical outcomes. This study aimed to investigate the distribution characteristics of five important OMP genes (*sabA*, *hofC*, *homA*, *homB* and *frpB-4*) in different regions of China.

**Materials and method:**

A total of 266 strains were isolated from 348 stomach biopsy specimens in Shandong, Guangxi, Heilongjiang, Hunan, and Qinghai provinces. The presence of *sabA*, *hofC*, *homA*, *homB* and *frpB-4* gene was detected by polymerase chain reaction (PCR) from *H*. *pylori* genomic DNA.

**Results:**

Among the strains in five regions, the prevalence of *frpB-4* was 100% and that of *hofC* was 97.7%. The prevalence of *homB* in the isolates from Qinghai (45.5%) was significantly lower than that in Shandong (75.3%), Guangxi (76.9%) and Hunan (69.6%) (*P*<0.05). The frequency of *homA* in Shandong (30.1%) was significantly lower than in Guangxi (57.7%) and Qinghai (63.6%) (*P*<0.05). The prevalence of the *sabA* gene in Shandong, Guangxi, Heilongjiang, Hunan and Qinghai provinces was 21.9%, 59.7%, 45.9%, 52.2%, and 18.2%, respectively (*P*<0.05). The *sabA* “on” status was significantly more frequent in isolates from Guangxi (46.8%), Heilongjiang (37.8%), and Hunan (47.8%) than Qinghai (3.0%) (*P*<0.05). The presence of *homA* and *sabA* genes may be negatively correlated with the development of gastritis. There was no significant association between the *frpB-4*, *hofC*, *homB* gene and clinical outcomes.

**Conclusion:**

The prevalence of *homA*, *homB*, and *sabA* genes and the *sabA* “on” or “off” status have significant geographical differences among five provinces in China. The presence of *homA* and *sabA* genes may be protective factors of gastritis.

## Introduction

*Helicobacter pylori* (*H*. *pylori*) is a Gram-negative, helix-shaped, microaerophilic, flagellated bacterium, which colonizes the mucus layer of the gastric epithelium [1]. *H*. *pylori* infection leads to peptic ulcer disease (PUD) in 15%-20% of the infected population, dyspepsia in 5%-10% of the infected population, and gastric cancer (GC) or mucosa-associated lymphoid tissue (MALT) lymphoma in 1% of the infected population [2]. While the exact molecular mechanisms by which *H*. *pylori* infection induces a severe clinical outcome have not yet been clearly elucidated, they are thought to involve various elements, including host genetic and environmental factors, as well as certain bacterial virulence genes [3].

The outer membrane is the outer barrier of Gram-negative bacteria, which consists of two highly asymmetric layers: the inner monolayer contains only phospholipids and the outer monolayer consists mainly of outer membrane proteins (OMPs) that are resistant to the external environment [4, 5]. About 4% of *H*. *pylori* genome encodes OMP [6]. According to their functions, they can be divided into five homologous gene families [6]: Hop (outer membrane porins) and Hor (Hop-related proteins) proteins, Hof (H. pylori OMP) proteins, Hom (*H*. *pylori* outer membrane) proteins, iron-regulated OMPs, and efflux pump OMPs families.

HopP belongs to the Hop family, also known as sialic acid-binding adhesion (SabA). SabA specifically binds to sLex antigen on gastric mucosa epithelial cell and plays an important role in *H*. *pylori* colonization and inflammation mediation [7, 8]. SabA expression level can quickly adapt to changes in the human gastric environment by “on” or “off”. The “on” status of *sabA* was negatively correlated with the degree of gastric acid secretion, suggesting that the pH or antigen expression of atrophic mucosa may affect the expression of SabA.

HofC is a member of a paralogous Hof family which consists of eight members. HofC encodes a non-thermal denatured protein with 528 amino acid residues, which has a hydrophobic C-terminal sequence motif of many outer membrane proteins [6]. HofC proteins are involved in passive diffusion and adhesion of cations such as antibiotics [9]. The *hofC* gene is highly variable in global strains and shows many America-differentiated SNPs and region-differentiated SNPs within the Americas [10].

Hom family is a small OMP family composed of HomA, HomB, HomC, and HomD. The *homA* gene, which presents more than 90% identity to *homB* [6]. Interestingly, *homA* was more frequently found in strains isolated from non-ulcer dyspepsia (NUD). In vitro, HomB promotes the secretion of the interleukin-8 (IL-8) and increases *H*. *pylori* adhesion. The *homA* and *homB* sequences have considerable geographic heterogeneity [11]. The prevalence of the *homA* and *homB* genes is different in strains all over the world, and there are significant differences between East Asian and Western strains [12].

*H*. *pylori* contains six iron-regulated OMPs. These OMPs can be divided into two groups based on homology. One of them is homologous of the Neisseria gonorrhoeae ferric enterobactin receptor FrpB, encoded by *frpB-1*, *frpB-2/3*, and *frpB-4* [6, 13]. A gene containing multiple highly differentiated SNPs is *frpB-4*, encoding for the first TonB-dependent nickel transport system across a bacterial outer membrane [14]. Studies have shown that the protein encoded by *frpB-4* will show specific amino acid changes in different regions, and these changes may affect the transport of nickel, thereby affecting the activity of urease [10].

China is a country with a vast territory and a large population. The five provinces selected in this study cover the eastern (Shandong), northern (Heilongjiang), western (Qinghai), southern (Guangxi), and central (Hunan) regions of China. The current infection rate of *H*. *pylori* in China is about 40–50%. *H*. *pylori* is one of the causes of gastric cancer (GC), which is the second most common cancer in China. Shandong (30.50/100000) and Qinghai (48.76/100000) provinces are considered to have a high incidence of GC, while Heilongjiang (15.53/100000), Guangxi (19.68/100000) and Hunan (10-20/100000) provinces have a relatively low incidence of GC [15, 16]. Meanwhile, the economic development level, living environment and dietary habits of these selected regions are quite different, which is more helpful for us to analyze the diversity of *H*. *pylori* distribution in China. At present, studies on OMP pay more attention to its correlation with diseases and other mechanisms. Only a few studies have analyzed the differential characteristics of *H*. *pylori* OMP coding genes in different regions. Therefore, we studied the distribution characteristics of *sabA*, *hofC*, *homA*, *homB*, and *frpB-4* in different regions of China and their association with clinical outcomes.

## Materials and methods

### Study subjects

A total of 266 patients were included in this study, including 73 cases in Weihai, Shandong province, 77 cases in Nanning, Guangxi province, 46 cases in Yiyang, Hunan province, 37 cases in Jiamusi, Heilongjiang province, and 33 cases in Haidong, Qinghai province, as previously reported [17]. Endoscopic examination showed PUD in 15 patients, chronic superficial gastritis (CSG) in 81 patients, chronic erosive gastritis (CEG) in 87 patients, chronic atrophic gastritis (CAG) in 42 patients. Their gastric biopsy specimens were obtained during upper gastrointestinal endoscopy with informed consent. This study was approved by Ethical Committee of National Institute for Communicable Disease Control and Prevention Chinese Center for Disease Control and Prevention (approval No. ICDC-2013001).

### *H*. *pylori* culture and identification

The strains were obtained from patients with varying gastric diseases as diagnosed by routine endoscopy and biopsy sampling. *H*. *pylori* strains were cultured on Karmali agar base plates supplemented with 5% defibering sheep blood and 1% combined antibiotics. The plates were grown at 37°C under microaerobic conditions (5% O_2_, 10% CO_2_ and 85% N_2_) approximately for 3–5 days. The organisms were identified as *H*. *pylori* based on colony morphology and Gram staining, as well as positive reactions in oxidase, catalase and urease biochemical tests, and also subsequently the results of *H*. *pylori*-specific polymerase chain reaction (PCR). All isolates were maintained at -80°C in sterile brain heart infusion (BHI) broth with 20% glycerol.

### DNA isolation and PCR amplification

Genomic DNA was extracted by QIAamp DNA Mini Kit according to the manual instruction. The DNA was stored at -20°C for molecular studies. All PCR amplification reactions were performed by standard methods in a final volume of 25 μL containing forward and reverse primers (0.2 μM each), 2 ng/μL DNA template, 12.5 μL 2×EasyTaq PCR SuperMix (Transgen, China) and 9.5 μL nuclease-free water. The PCR protocol (35 cycles) included a denaturing step at 94°C for 30 sec, annealing at 55, 56, 55, 55 and 60°C for *hofC*, *frpB-4*, *homB*, *homA* and *sabA*, respectively, for 30 sec, and extension at 72°C for 1 minutes followed by a 10-minute extension step at 72°C. The *sabA* gene was detected by PCR, which was additionally sequenced to define its functional status as either “on” or “off”. The nucleotide sequences of the *sabA* gene were submitted to China National Microbiological Data Center. The accession numbers are NMDCN0000R8M to NMDCN0000RBU. The functional status of *sabA* is regulated by the number of CT-dinucleotide repeats. The information of the PCR primer for each amplicon is summarized in Table 1. PCR products were then electrophoresed for 35 min at 130 Volt on 1.5% agarose gel in the presence of Gelstain and illuminated by the gel documentation system (Bio-Rad, USA).

**Table 1:** Primers used for PCR amplification of *hofC*, *frpB-4*, *homB*, *homA*, and *sabA* genes.

Gene	Primer	primer sequence (5’→3’)	Product size (bp)
*hofC*	*hofC*-F	GCTTGCCACTRTTGTTCACT	907
	*hofC*-R	CGACCGTATTCAGCGTTATT	
*frpB-4* [18]	*frpB-4*-F	AGCCGTCTCTTAAGGGTAAC	407
	*frpB-4*-R	TCGCTATTGCTTGGATCTTG	
*homB* [19]	*homB*-F	AGAGGGTGTTTGAAACGCTCAATA	161
	*homB*-R	GGTGAATTCTTCTGCGGTTTG	
*homA* [19]	*homA*-F	AGAGGGTGTTTGAAACGCTCAATA	128
	*homA*-R	GGTGAATTCTTCTGCGGTTTG	
*sabA* [20]	*sabA*-F	GTGGATCCCTTTAAGGAACATTTTATGAAAA	600
	*sabA*-R	GGAATTCGGTTAGGATAAAAGCGCAAAGATTGT	

### DNA sequencing

Samples without target genes of *sabA* were discarded at the identification stage of the product. The positive PCR products of isolates were purified and then the DNA samples were submitted and performed as a service by the Beijing Genomics Institute for the sequence of *sabA* genes. The *sabA* gene resulting PCR products were used for DNA sequencing to determine the number of CT-dinucleotide repeats.

### Statistical analysis

Statistical analysis was performed by chi-squared test for independence. All data analysis were performed using the SPSS software version 25. All tests of significance were two-tailed with a *P*-value<0.05 taken as significant.

## Result

A total of 266 *H*. *pylori* isolates from five geographic regions of China were obtained, of which 73 isolates were from Shandong, 77 from Guangxi, 46 from Hunan, 33 from Qinghai and 37 from Heilongjiang. The *hofC*, *frpB-4*, *homB*, *homA* and *sabA* genes were detected by PCR in all isolates and the results were summarized in Table 2.

**Table 2:** Distribution of OMP genes among 267 *H*. *pylori*-positive patients with different geographic regions.

Genetypes	Shandong	Guangxi	Heilongjiang	Hunan	Qinghai	Total	χ^2^	*P*-value
	(n = 73)	(n = 77)	(n = 37)	(n = 46)	(n = 33)	(n = 266)		
*hofC* ^+^	70 (95.9)*	77 (100)	37 (100)	45 (97.8)	31 (93.9)	260 (97.7)	5.937	0.204
*hofC* ^-^	3 (4.1)	0	0	1 (2.2)	2 (6.1)	6 (2.2)		
*frpB-4* ^+^	73 (100)	77 (100)	37 (100)	46 (100)	33 (100)	266 (100)		
*homB* ^+^	55 (75.3)	59 (76.6)	23 (62.2)	32 (69.6)	15 (45.5)	184 (69.2)	12.870	0.012
*homB* ^-^	18 (24.7)	18 (23.4)	14 (37.8)	14 (30.4)	18 (54.5)	82 (30.8)		
*homA* ^+^	22 (30.1)	44 (57.1)	17 (45.9)	19 (41.3)	21 (63.6)	123 (46.2)	15.766	0.003
*homA* ^-^	51 (69.9)	33 (42.9)	20 (54.1)	27 (58.7)	12 (36.4)	143 (53.8)		
*sabA* ^+^	16 (21.9)	46 (59.7)	17 (45.9)	24 (52.2)	6 (18.2)	109 (41.0)	32.024	<0.001
*sabA* ^-^	57 (78.1)	32 (40.3)	20 (54.1)	22 (47.8)	27 (81.8)	158 (59.0)		
*sabA*“on”	11 (15.1)	36 (46.8)	14 (37.8)	22 (47.8)	1 (3.0)	84 (31.6)	16.210	0.003
*sabA*“off”	5 (6.8)	10 (13.0)	3 (8.1)	2 (4.3)	5 (15.2)	25 (9.4)		

*Values in parentheses are percentages

### *hofC* status and *frpB-4* status

The 907-bp PCR product indicating the presence of *hofC* gene of *H*. *pylori* was determined in 260 patients (97.7%), whereas 6 (2.2%) patients were classified as *hofC*-negative. Of these *hofC*-positive strains, 70 (95.9%) strains were isolated from Shandong, 45 (97.8%) from Hunan, 31 (93.9%) from Qinghai, 100.0% from Guangxi and Heilongjiang. There were no significant differences between the *hofC* gene and clinical outcomes (χ^2^ = 5.937, *P*>0.05). The *frpB-4* gene was found in all 266 (100.0%) isolates.

### *homB* and *homA* status

The *homB* gene was determined in 184 isolates (69.2%), whereas 82 isolates (30.8%) were classified as *homB*-negative. The prevalence of *homB* in the isolates from Qinghai (45.5%) (χ^2^ = 12.870, *P*<0.05) was significantly lower than that in Shandong (75.3%), Guangxi (76.6%), and Hunan (69.6%). For another hom family gene, 123 (46.2%) patients were infected with *homA* and 143 (53.8%) patients were *homA*-negative. The frequency of *homA* in Shandong (30.1%) (χ^2^ = 15.766, *P*<0.05) was significantly lower than in Guangxi (57.1%) and Qinghai (63.6%). There was no association between the *homB* genes and clinical outcomes in all five regions. However, in the strain from all provinces, *homA* showed significant differences between CSG and CAG, suggesting that *homA* may be associated with the severity of gastritis, as shown in S1 Table (χ^2^ = 6.707, *P*<0.05). The presence of *homA* may be a protective factor for gastritis (OR = 0.364).

### *sabA* status

Sixteen *sabA* genes from Shandong province, 46 from Guangxi province, 17 from Heilongjiang province, 24 from Hunan province, 6 from Qinghai province contained a run of CT repeats. The *sabA* frequency was significantly more prevalent in Guangxi (59.7%), Hunan (52.2%), and Heilongjiang (45.9%) than in Shandong (21.9%) and Qinghai (18.2%) (χ^2^ = 32.024, *P*<0.001). Moreover, the presence of the *sabA* gene in *H*. *pylori* isolates was correlated with the severity of gastritis. The severity of CSG, CEG, and CAG increased successively. The *sabA*-positive gene was significantly related to the differences of CSG and CAG (χ^2^ = 10.539, *P*<0.05). The *sabA* gene was negatively correlated with the development of gastritis (OR = 0.253).

All *sabA* genes tested displayed different CT-repeat lengths ranging from 2 to 10 repeats between different *H*. *pylori* strains. In all cases, the CT repeats were associated with the 5’ region of *sabA* genes. In addition, the repeated pattern of CT dinucleotide in the coding region was found and classified. In 109 *sabA-*positive gene sequencing results, 84 (77.1%) of which had a switch on status while 25 (22.9%) had an off status. The *sabA* functional status was significantly more frequent in isolates from Guangxi (46.8%), Heilongjiang (37.8%), and Hunan (47.8%) than Qinghai (3.0%) (χ^2^ = 16.210, *P*<0.05). Furthermore, the repeated pattern of CT dinucleotide varied in the sequences which were different from *sabA* (Table 3). The pattern containing 2 CT repeats was the most frequently associated with the “on” status (58/109, 53.2%), and the pattern with 6 CT repeats was the most prevalent for a nonfunctional (“off”) *sabA* gene (9/25, 36%). However, the differences between the *sabA* status and clinical outcome were not statistically significant.

**Table 3:** Frequency of the *sabA* CT repeats patterns.

Strain	CT repeat	Sequence of the signal peptide coding region	Amino acid sequence	*sabA* status	Number(%)
HP-1	2	ATG-CTCTAT-CTGAAGATA-TTTTTG**TGA**	MKK-LSLSL-FL*	Off	1 (0.9)
HP-2	2	ATG-CTCTAT-GCTGAAGAC-TTTTTTGTGAGC	MKK-LSL-AED-FFVS	On	58 (53.2)
HP-3	4	ATG-CTCTCTCTCG-GCTGAAGAT-TTTTTTGTGAGC	MKK-LSL-AED-FFVS	On	9 (8.3)
HP-4	5	ATG-CTCTCTCTCTCG-TTT**TAA**	MKK-LSLSL-F*	Off	3 (2.8)
HP-5	5	ATG-CTCTCTCTCTCG-GCTGAAGAC-TTTTTTGTGAGC	MKK-LSLSL-AED-FFVS	On	4 (3.7)
HP-6	6	ATG-CTCTCTCTCTCTCGC-GCACGC**TGA**	MKK-LSLS-AR*	Off	9 (8.3)
HP-7	7	ATG-CTCTCTCTCTCTCTCG-CGC**TGA**	MKK-LSLSL-R*	Off	3 (2.8)
HP-8	7	ATG-CTCTCTCTCTCTCTCG-GCTGAAGAC-TTTTTTGTGAGC	MKK-LSLSL-AED-FFVS	On	6 (5.5)
HP-9	8	ATG-CTCTCTCTCTCTCTCTCG-ACGCTGAAG-TTTTTG**TGA**	MKK-LSLSLSL-FL*	Off	3 (2.8)
HP-10	8	ATG-CTCTCTCTCTCTCTCTCG-ACGCTGAAG-TTTTTGTGA	MKK-LSLSLSL-FFVS	On	6 (5.5)
HP-11	9	ATG-CTCTCTCTCTCTCTCTCTCG-TTTTTA**TGA**	MKK-LSLSLS-FL*	Off	5 (4.6)
HP-12	10	ATG-CTCTCTCTCTCTCTCTCTCTCG-GCTGAAGAC-TTTTTTATAAGC	MKK-LSLSLSL-AED-FFIS	On	1 (0.9)
HP-13	10	ATG-CTCTCTCTCTCTCTCTCTCTCGCT-CGC**TGA**	MKK-LSLSLSL-R*	Off	1 (0.9)

*Indicates stop codon

### Combined presence of OMP encoding genes *frpB-4*, *hofC*, *homB*, *homA*, and *sabA* in different regions

The presence of *homA* was associated with the presence of *homB* (χ^2^ = 124.332, *P*<0.001) and *sabA* (χ^2^ = 5.482, *P*<0.05). A statistically significant correlation between *homA* and *homB* and *sabA* gene was detected. The relationship between *homA* and *homB* is even closer (r = 0.564) (Table 4).

**Table 4:** Comparison and statistical analysis of *H*. *pylori* OMP genes.

Comparison	χ^2^	*P-*value	r
*hofC* vs *frpB-4*	/	/	
*hofC* vs *homB*	0.338	0.561	0.063
*hofC* vs *homA*	0.361	0.548	0.062
*hofC* vs *sabA*	0.0E0	1.000	0.024
*frpB-4* vs *homB*	/	/	/
*frpB-4* vs *homA*	/	/	/
*frpB-4* vs *sabA*	/	/	/
*homB* vs *homA*	**125.597** *	**3.7671E** ^**-29**^	0.566
*homB* vs *sabA*	2.984	0.084	0.105
*homA* vs *sabA*	**5.760**	**0.016**	0.146

*Numbers in boldface type indicate a significant correlation between the two genes

As *frpB-4* and *hofC* genes were almost all positive in all 266 strains, and the correlation between the two genes and geographical distribution was not statistically significant, we selected strains that were both positive for *frpB-4* and *hofC* when analyzing the association between gene combination and geographical origin, and also excluded confounding factors.

When considered in five genes associated combinations (Table 5), the frequency of *hofC*/*frpB-4*/*homB*/*homA* genotypes and *hofC*/*frpB-4*/*homB*/*homA*/*sabA* genotypes was significantly in Guangxi (33.8%) (χ^2^ = 27.364, *P*<0.001 and χ^2^ = 39.397, *P*<0.001) higher compared to the other four provinces. The frequency of *hofC*/*frpB-4*/*homB*/*sabA* genotypes in Shandong, Guangxi, Heilongjiang, Hunan and Qinghai provinces were 12.3%, 44.2%, 18.9%, 37.0%, 3.0%, respectively (χ^2^ = 33.509, *P*<0.001). Furthermore, the *hofC*/*frpB-4*/*homA*/*sabA* genotypes frequency was significantly more prevalent in Guangxi (39.0%) (χ^2^ = 24.127, *P*<0.001) than in Shandong (8.2%), Hunan (17.4%) and Qinghai (12.1%). The *hofC*/*frpB-4*/*homA*/*sabA* genotypes were significantly more frequent in Heilongjiang (27.0%) (χ^2^ = 24.127, *P*<0.001) than in Shandong (8.2%).

**Table 5:** Prevalence of combined *H*. *pylori hofC*, *frpB-4*, *homA*, *homB*, *sabA* in different regions.

Genetype	Shandong	Guangxi	Heilongjiang	Hunan	Qinghai	Total	χ^2^	*P*-value
	(n = 73)	(n = 77)	(n = 37)	(n = 46)	(n = 33)	(n = 266)		
*hofC*^+^&*frpB-4*^+^&*homB*^+^&*homA*^+^*	4 (5.5)	27 (33.8)	3 (6.8)	5 (10.9)	4 (12.1)	42 (15.8)	27.364	<0.001
*hofC*^-^|*frpB-4*^-^|*homB*^-^|*homA*^-^*	69 (94.5)	51 (66.2)	34 (91.9)	41 (89.1)	29 (87.9)	224 (84.2)		
*hofC*^+^&*frpB-4*^+^&*homB*^+^&*sabA*^+^	9 (12.3)	34 (44.2)	7 (18.9)	17 (37.0)	1 (3.0)	68 (25.6)	33.509	<0.001
*hofC*^-^|*frpB-4*^-^|*homB*^-^|*sabA*^-^	64 (87.7)	43 (55.8)	30 (81.1)	29 (63.0)	32 (97.0)	198 (74.4)		
*hofC*^+^&*frpB-4*^+^&*homA*^+^&*sabA*^+^	6 (8.2)	30 (39.0)	10 (27.0)	8 (17.4)	4 (12.1)	58 (21.8)	24.127	<0.001
*hofC*^-^|*frpB-4*^-^|*homA*^-^|*sabA*^-^	67 (91.8)	47 (61.0)	27 (73.0)	38 (82.6)	29 (87.9)	208 (78.2)		
*hofC*^+^&*frpB-4*^+^&*homB*^+^&*homA*^+^&*sabA*^+^	1 (1.4)	18 (23.4)	0	1 (2.2)	0	20 (7.5)	39.397	<0.001
*hofC*^-^|*frpB-4*^-^|*homB*^-^|*homA*^-^|*sabA*^-^	72 (98.6)	59 (76.6)	37 (100)	45 (97.8)	33 (100)	246 (92.5)		

*‘&’ indicate ‘and’, ‘|’ indicate ‘or’, values in parentheses are percentages

The above four gene combinations were negatively correlated with gastritis, which may be protective factors of gastritis. The specific OR values are shown in S1 Table.

## Discussion

*H*. *pylori* is one of the most successful pathogens that colonizes the stomach of half the world’s population for a long time [21]. *H*. *pylori* infection can cause serious clinical consequences such as chronic gastritis, PUD, gastric atrophy, and GC [22, 23]. The prevalence of *H*. *pylori* depends on geographic regions, age, occupation, social and economic status, and living environment [24]. Outer membrane proteins are very important during infection and can influence the levels of bacterial colonization [25].

In this study, the distribution of *frpB-4*, *hofC*, *homB*, *homA*, and *sabA* genes in *H*. *pylori* isolated from patients suffering from gastric diseases in China was determined by PCR, and the relationship between these OMP encoding genes was assessed.

The results showed that there were significant statistical differences in the distribution of *homA*, *homB*, and *sabA* genes in different provinces of China. The distribution of different gene combinations in the five provinces was also showed significant statistical differences. This may be related to the selective adaptation of genes in different regions. China, as a country with vast territory, its geographical distribution of population has been in a relatively stable state for a long time, which may lead to *H*. *pylori* infection in different regions of China with more significant OMP distribution characteristics.

*H*. *pylori* carries two paralogous OMP, HomA and HomB, which have recently been suggested to be important determinants of disease severity [12]. In our study, the *homB* gene was significantly more prevalent than the *homA* gene (χ^2^ = 125.597, *P*<0.001). The positive rate of *homB* was the highest in Shandong province and the lowest in Qinghai Province, while *homA* was the opposite. The presence of the *homA* gene was significantly associated with the absence of *homB* gene since only 43 (16.2%) simultaneously harbored both genes. In western countries, the prevalence of *homA* was 61.9% and that of *homB* was 61.2%, which were approximately the same. There was no significant difference in *homB* prevalence between Colombian and American strains [12]. Compared with our result, the prevalence of *homA* (46.2%) was significantly different. Another study showed that *homA* and *homB* genes were heterogeneously distributed worldwide, with significant differences between East Asian and Western strains. Moreover, *homB* was found more frequently in East Asian strains than *homA*, but were not associated with clinical outcome. The presence of both genes in the same genome was detected in 10.4% of strains [26]. This is consistent with the results of our study. In addition, it was also pointed out that the copy number polymorphism of *homB* and *homA* had obvious geographical specificity [26]. Further sequence analysis using *H*. *pylori* strains from different geographic backgrounds in this study can assess whether different alleles could be associated with the severity of clinical outcomes or different geographic origin [26, 27].

In this study, the prevalence of *sabA*-positive gene was 41.0% (109/266) and that of *sabA* “on” was 31.6% (84/266). The prevalence of Portugal, the Netherlands, and Italy were 63.2%, 49.0%, and 35.5%, respectively [28–30]. The *sabA*-positive strains accounted for 85.3% of the Iranian studies, but the prevalence of *sabA* “on” *status* was not reported [31]. In contrast, the prevalence of *sabA* functional status was higher in Japan (81.5%) [20]. Analysis from Taiwan showed that 80.0% of strains had the *sabA* gene, while only 31.0% (45/145) strains carried *sabA* [8]. These large differences may be due to different disease sources of the strains or genetic diversity in different parts of the world. In addition, the association between *sabA* functional status and disease has not been fully established, and there is significant geographical diversity.

In this study, 100% of *H*. *pylori* strains carried the *frpB-4* gene and 97.7% carried the *hofC* gene, which has nothing to do with geographic origin and clinical outcome. Specific positive rates of *frpB-4* and *hofC* in different regions have not been reported. However, it was found that *frpB-4* was a gene containing multiple highly differentiated SNPs in strains from different regions of the world, especially in northeast China. The *hofC* gene is highly variable in global strains and shows many America-differentiated SNPs and region-differentiated SNPs within the Americas [9]. In Fujian, China, an area with a high incidence of cancer, HofC also contains the most differentiated SNPs [10, 32]. Therefore, we should analyze the relationship between the two and the disease and geographic distribution from a more microscopic perspective.

There are still some limitations to our research. At present, we only studied the distribution characteristics of *H*. *pylori* outer membrane protein encoding genes in different regions of China, mainly comparing the positive rate of genes in different regions, gene interaction, genotype and gene combination with regional distribution and clinical outcomes. We have not thoroughly studied the function and mechanism of one or several genes in *H*. *pylori* colonization and pathogenicity. We will supplement it in the following study.

## Conclusion

The *homA*, *homB* and *sabA* genes of *H*. *pylori* have significant geographical differences among five provinces in China. The *homA* and *sabA* genes were negatively correlated with the severity of gastritis.

## Supporting information

S1 Table Frequency of 267 *H*. *pylori* OMP genes in patient with CSG, CEG and CAG. (DOCX)
