# Supplementary material for: Distribution characteristics of the sabA, hofC, homA, homB and frpB-4 genes of Helicobacter pylori in different regions of China
Source: PLoS One. 2022 May 19;17(5):e0268373. doi: 10.1371/journal.pone.0268373 (PMC9119684; doi:10.1371/journal.pone.0268373)
Supplement: S1 Table — (DOCX) [file pone.0268373.s001.docx]

Table S1 Frequency of 267 *H. pylori* OMP genes and gene combinations in patients with CSG, CEG, and CAG

*Values in parentheses are percentages. Numbers in boldface type indicate a significant correlation between the gene and disease. CSG: chronic superficial gastritis, CEG: chronic erosive gastritis, CAG: chronic atrophic gastritis

| Gene | No. of isolates | | | |  |  |  |  |
| --- | --- | --- | --- | --- | --- | --- | --- | --- |
|  | Total (n=210) | | | χ^2^ | P-value | r | OR | 95%CI |
|  | CSG (n=81) | CEG (n=87) | CAG (n=42) |  |  |  |  |  |
| *hofC^+^* | 80 (98.8)* | 85 (97.7) | 40 (95.2) | 1.485 | 0.476 | 0.084 |  |  |
| *frpB-4^+^* | 81 (100) | 87 (100) | 42 (100) | \ | \ | \ |  |  |
| *homB^+^* | 62 (76.5) | 58 (66.7) | 31 (73.8) | 2.120 | 0.347 | 0.100 |  |  |
| *homA^+^* | 40 (49.4) | 41 (47.1) | 11 (26.2) | **6.707** | **0.035** | 0.176 | 0.364 | 0.161 to 0.821 |
| *sabA^+^* | 42 (51.9) | 37 (42.5) | 9 (21.4) | **10.539** | **0.005** | 0.219 | 0.253 | 0.108 to 0.596 |
| *sabA*“on” | 33 (40.7) | 30 (34.5) | 8 (19.0) | 0.513 | 0.774 | 0.076 |  |  |
| *sabA*“off” | 9 (11.1) | 7 (8.0) | 1 (2.4) |  |  |  |  |  |
| *hofC*+&frpB4+&homB+&homA+ | 21 (25.9) | 11 (12.6) | 2 (4.8) | **10.507** | **0.005** | 0.218 | 0.414 | 0.185 to 0.924 |
| *hofC^+^&frpB4^+^&homB^+^&sabA^+^* | 31 (38.3) | 24 (27.6) | 4 (9.5) | **11.334** | **0.003** | 0.226 | 0.17 | 0.055 to 0.522 |
| *hofC^+^&frpB4^+^&homA^+^&sabA^+^* | 23 (28.4) | 16 (18.4) | 4 (9.5) | **6.446** | **0.04** | 0.173 | 0.265 | 0.085 to 0.828 |
| *hofC^+^&frpB4^+^&homB^+^&homA^+^&sabA^+^* | 13 (16) | 4 (4.6) | 0 | **12.018** | **0.002** | 0.233 | 0.252 | 0.079 to 0.809 |
